# Developing teachers’ competency for inclusive education in Ethiopia

**DOI:** 10.4102/ajod.v13i0.1383

**Published:** 2024-12-20

**Authors:** Aschalew T. Kebede, Tlakale N. Phasha

**Affiliations:** 1 Centre of Excellence in Disabilities, Faculty of Education, University of South Africa, Pretoria, South Africa

**Keywords:** mixed method, inclusive pedagogy, teacher training, inclusion, inclusive education, competency, Ethiopia

## Abstract

**Background:**

In light of the increasing diversity within school populations globally, a pressing need arises for nations to prioritise the enhancement of teachers’ competencies in implementing inclusive education, recognising the central role teacher education programmes play in addressing this imperative. Teacher education programmes centred on inclusion not only respond to this global priority but also wield influence on teachers’ attitudes, self-efficacy and stress levels when engaging with diverse learners.

**Objectives:**

The study presents an analysis of the effectiveness of a teacher education programme in Ethiopia in developing teachers’ competencies for inclusion.

**Method:**

Using a sequential explanatory mixed-methods research design, involving 159 teachers, data were collected through questionnaires (*n* = 152) and individual interviews (*n* = 7). Quantitative data were analysed using SPSS, and qualitative data underwent thematic analysis. This approach ensures a nuanced examination of the research question with precision and depth.

**Results:**

Findings highlight challenges in the Ethiopian teacher education system, revealing a significant lack of provision of sufficient knowledge to empower teachers to understand inclusion. The inadequacy extends to the influence on teachers’ attitudes towards inclusion, as well as the insufficient exposure to practical, hands-on experiences essential for addressing diverse learning needs.

**Conclusion:**

The challenges faced by teacher training in Ethiopia, evident in its struggle to align with global standards for supporting teachers in implementing inclusive education, necessitate urgent and substantial reforms.

**Contribution:**

Addressing the gaps in understanding inclusion, fostering positive attitudes and enhancing practical experiences necessitating a comprehensive overhaul of teacher education curricula and practices.

## Introduction

With schools becoming increasingly diverse in terms of learner populations, it becomes imperative for countries to prioritise the development of teachers’ competencies in implementing inclusive education. In responding to this global priority, teacher education programmes are emerging as significant means to foster these essential skills. Previous studies have indicated how a teacher education programme focussing on inclusion has a significant impact on teachers’ attitudes towards inclusive education, their self-efficacy and confidence, as well as reducing their stress levels in relation to dealing with diverse learners (Cardona 2009; Chamber & Forlin 2010; Coates 2012; Issaka, Nyaaba & Iddrisu 2022; Kim 2013; Symeonidou & Phtiaka 2014; World Bank 2013). These programmes also contribute to teachers’ positive values and ethical understandings to ensure an inclusive environment in their classrooms (Forlin 2012). Such teacher training programmes facilitate the development of core competencies such as knowledge, practical skills and attitudes to successfully implement inclusive education. However, Darling-Hammond (2017) suggests that teacher education programmes that train teachers for inclusion need re-evaluation to ensure their efficacy in knowing, being, and doing, as there is limited evidence to judge their effectiveness in preparing teachers for the successful implementation of inclusive education in many countries, including Ethiopia.

The Ethiopian government’s commitment to promoting inclusive education is evident in its educational policies, which have been informed by international declarations and conventions ratified since 1994. These policies focus on promoting the holistic development of children, including those with disabilities, orphans, homeless and working children, by protecting them from diseases and abuse and creating an optimal learning environment (EMoE 2015). Furthermore, the government implemented the *Education and Training Policy* in 1994, which focusses on expanding general education for girls and women as well as students with disabilities (World Bank 2013). However, the policy’s main goals, namely, access, equity, quality and relevance, have been overshadowed by a push to increase enrolment. To address this problem, the government has introduced teacher development programmes focussing on professional competencies and promoting inclusion in education (EMoE 2012; World Bank 2013). The programme is expected to develop teachers with the necessary knowledge, skills and beliefs necessary for their role as inclusive practitioners by the end of the training (EMoE 2011). However, there are few studies that have investigated the views of teachers on whether the Postgraduate Diploma in Teaching (PGDT) has supported them in developing competencies necessary for inclusion and becoming agents in Ethiopia. This is critical as teacher education programmes are essential in building knowledge, attitudes and practical skills that facilitate the effective implementation of inclusion in education. In this context, the study’s aim is to investigate the influence of teacher training programmes on teachers’ knowledge, attitudes and practical skills in implementing inclusive pedagogy for learners with disabilities in Ethiopia. By prioritising learners with disabilities, the article seeks to contribute to the creation of a more accessible and supportive educational environment that ensures that every learner has the opportunity to thrive. Specifically, the study addressed the question: To what extent does teacher preparation contribute to the development of essential competencies required for inclusive education in Ethiopia?

The Ethiopian government defines inclusion as a process that strengthens the capacity of the education system to reach all learners, guided by the principle that education is a fundamental right and the cornerstone of a just and equitable society (EMoE 2017). Inclusive education encompasses an education system that welcomes all learners, irrespective of poverty, gender, ethnicity, language, disabilities or impairments. This approach emphasises that all learners can learn, and it places particular emphasis on supporting groups of learners who are at risk of marginalisation, exclusion or academic underachievement (EMoE 2017). Therefore, the competency of teachers explored in this article is for the inclusion of all learners experiencing various barriers to learning in secondary school classrooms in Ethiopia, including learners with disabilities. Having said that, the section that follows will clarify the core competencies for inclusive education.

### Core competencies necessary for inclusion in education

Although the definition of these competencies is influenced by national standards, various authors have identified practical skills, knowledge and beliefs as fundamental competencies for inclusive classrooms (Fisher, Frey & Thousand 2003; Hick et al. 2019; Johnstone & Chapman 2009; Liakopoulou 2011). In the ‘Knowledge-related competency’ section, we elaborate on each of these competencies.

### Knowledge-related competency

Teachers’ knowledge about inclusion and barriers to learning, including those affecting students with disabilities, is important for the successful implementation of inclusive education. Studies suggest four major areas of teacher knowledge for inclusion: content knowledge, pedagogical knowledge, knowledge of each student’s abilities and disabilities, learning strengths and needs, prior experiences, cultural and linguistic backgrounds, specialised knowledge of specific disabilities and contextual knowledge of inclusive education policies, procedures and legal requirements (Bocala et al. 2010; Kortjass 2012; Le Page et al. 2010; Mu, Wang & Wang 2015; Sanches-Ferreira 2012). These areas help teachers carry out their work effectively and cater to divese needs (Mu et al. 2015; Sanches-Ferreira 2012). In the same vein, Bocala et al. (2010) propose a comprehensive knowledge package for general education teachers working in inclusive contexts, which includes an understanding of the legal and historical foundations of inclusive education, the growth and development of children with special educational needs, and instructional design, planning and methods. Additionally, Kortjass (2012) identifies seven categories of teacher knowledge for the effective implementation of inclusive education: content, pedagogy, curriculum, learners and learning, schooling contexts, pedagogical content knowledge and educational philosophies.

However, some studies show teachers have a shallow understanding of inclusive education, highlighting the tension between desirable knowledge and reality (Jenkins & Ornelles 2009). As noted by LePage et al. (2010), teacher education programmes should expose candidates to the principles and philosophies of inclusive education, providing them with essential knowledge to develop a deep understanding of teaching and learning in inclusive settings. This knowledge includes content, professionalism, cross-cutting, emerging issues and practical understanding (Tabot & Osman 2017), as well as how to identify the needs and barriers to learning so that relevant pedagogic strategies can be applied. Thus, it is crucial for teacher trainees to have knowledge of inclusive education to develop effective classroom activities and understand a child’s disability, thereby promoting personal and social adjustment. This knowledge is essential for teachers working in inclusive settings (Opertti 2010).

### Attitudes-related competency

Teachers’ attitudes towards inclusion and students with disabilities are crucial for creating inclusive schools and communities. They promote inclusive practices (Forlin 2010), shape teachers’ perceptions of the social and physical world, and influence overt behaviours (Albarracín et al. 2008). Therefore, positive attitudes among teachers can create conducive learning environments and foster a sense of belonging (Sepadi 2018). In this regard, Ainscow, Booth and Dyson (2006) state that teaching is ineffective if teachers perceive themselves as lacking or fixed. Loreman, Sharma and Forlin (2013) maintain that teacher education is a context where changes in attitude towards inclusion occur. Similarly, Rouse (2010) notes that interventions aimed at helping teachers understand and implement inclusion need to address not only knowledge and skills but also attitudes towards inclusion and children with diverse educational needs. Therefore, affecting teachers’ attitudes is of great importance and is more beneficial during their teacher education programme (Tubele 2010).

Pearson (2007) also emphasises the importance of inclusive education training, which should include activities that positively change teachers’ attitudes. On the other hand, Isaaka, Nyaaba and Iddrisu (2022) showcase the effectiveness of a teacher training curriculum that explicitly outlines inclusive education as its foundational pillar. Similarly, Munyungu (2015) suggests that a gap between theory and practice is necessary for teachers to develop a positive attitude towards inclusivity. Above all, teacher training should provide opportunities for trainees to interact with diverse learners, policies and legislation related to inclusive education. Berisha and Vula (2021) also suggest that attitudes can be changed through comprehensive, well-structured and hands-on courses. Designing inclusive education training that benefits learners with diverse needs and the education system can help build an inclusive society (Sepadi 2018).

### Practical skills and abilities-related competency

Inclusive practices require teachers to develop key skills, including instructional and management abilities, to provide appropriate education for diverse student populations. Mu et al. (2015) suggest adequate preparation in these areas. In this regard, Idol (2006) identified three skill areas for the effective inclusion of students with disabilities: adaptation of instruction, curriculum modification, student discipline and classroom management. Similarly, Fisher et al. (2003) identified six essential skills for inclusive practices: collaborative teaming, curricular modifications, personal support, assistive technology, positive behavioural support and literacy instruction. Bocala et al. (2010) also recommended these skills for general education teachers, including adapting instructional methods, acquiring field experiences, preparing individualised education programmes, seeking support and identifying learning needs (Darling-Hammond 2006).

Research indicates that teacher training is the best time for teachers to acquire the necessary skills for inclusion (Ajuwon et al. 2012). Teachers’ perception of preparedness is linked to the skills and abilities they acquire during training and their responsibilities as inclusive teachers (Gorski 2012). Acquiring practical skills during training helps teachers teach better, plan lessons and address individual needs, which are essential for managing an inclusive classroom (Schunk 2008). Similarly, Mukhopadhyay, Molosiwa, & Moswela (2009) note that inclusive teachers are service providers in teaching students with diverse needs, and their skills contribute to their success or failure. Inadequate training can lead to discontent among students, affecting their confidence and success. Therefore, collaboration, student management, support and instructional accommodation are crucial practical skills for inclusive education teachers (Liakopoulou 2011).

New teachers often struggle with the complex demands of inclusive classrooms because of a lack of basic skills (Sosu, Mtika & Colucci-Gray 2010). In this regard, Darling-Hammond (2006) identified three fundamental skill-related problems: apprenticeship of observation, enactment and complexity. These problems require new teachers to understand teaching in different ways, act as teachers and respond to the dense and multifaceted nature of the classroom. To address these concerns, teacher preparation programmes should design courses that help prospective teachers appreciate environmental, social and cultural contexts of learning, behaviour, and teaching and practice these understandings in inclusive classrooms (eds. Alur & Timmons 2009).

This being the case, however, investigating the extent to which university-based teacher education programmes are effective in equipping teachers with the necessary competencies so that they can practice inclusion successfully is important. Teachers are expected to believe that they are qualified and capable of supporting all children in their teaching, boosting the capacity of all children to learn, and possess knowledge about socio-cultural perspectives on learning, as well as theoretical, policy and legislative issues of inclusion. In addition, they play a crucial role in creating favourable conditions for all children in an inclusive context. In this regard, the researcher adopted the framework developed by Rouse (2010) about ‘knowing’, ‘believing’ and ‘doing’ concerning inclusion and by Florian and Spratt (2013) concerning the basic principles of inclusive pedagogical approaches that include understanding learning, social justice and becoming an active professional, so as to check the extent to which the teacher education programme conducted in Ethiopia supports and equips teachers with the competencies necessary to implement inclusion in Ethiopian educational system. The ‘Methods’ section provides a detailed discussion of the methodology employed to conduct the present study.

## Methods

The research employed a mixed-methods approach, specifically adopting a sequential explanatory design as outlined by Creswell (2012). The integration of qualitative and quantitative data within mixed methods research is considered advantageous, leveraging their complementary strengths to address pertinent research inquiries most effectively (Grace 2014). The sequential explanatory design facilitated a two-phase data collection and analysis process, commencing with quantitative data in the initial phase and using its findings to inform the subsequent qualitative data collection and analysis phase.

### Research location

Haramaya University, located in Ethiopia’s eastern region, was chosen as a research site. As the oldest and largest public institution in Ethiopia, it has better resources and a strong track record in teacher training (EMoE 2018). The university has been offering the Postgraduate Diploma in Teaching (PGDT) programme since 2011.

### Sampling

Participants were selected from graduates who had attended the PGDT training at Haramaya University. They were qualified for secondary school teaching and held teaching posts in 12 secondary schools surrounding the university. Of a total of 173 participants who were invited to participate in the study, 152 indicated interest in participating. All 152 participants responded to the invitation, which was posted on the university’s notice boards, by contacting the first author directly through his mobile phone or email address.

The study used two sampling techniques to obtain participants – simple random and purposive. The techniques permitted the acquisition of both quantitative and qualitative data, thereby optimising the study’s ability to address the research questions comprehensively. Specifically, a simple random sampling approach was followed to obtain 152 (109 males and 49 females) teachers. This gender distribution revealed a notable imbalance, which may be attributed to historical gender equality in educational access in Ethiopia (EMoE 2015). Regarding age distribution, the majority of graduates (83%) fell within the 20–25 age bracket, followed by those in the 25–30 age range (15%). A minimal percentage (2%) comprised individuals aged 30–35, with no graduates exceeding the age of 35 in the study. The skew towards a younger demographic is aligned with Berger’s (2008) observation that the range of 20–30 is a critical period for educational pursuit and personal development.

Purposive sampling was followed to obtain participants to participate in the face-to-face, semi-structured interviews. They were seven in number – three females and four males. They had participated in the first phase of the study. Their ages ranged from 22 to 27. The teachers possessed different undergraduate degrees before undertaking the PGDT. These included Bachelor of Sciences degrees in Sports Science, Mathematics, Biology, Chemistry and Physical Sciences as well as Bachelor of Arts degrees in Geography, Environmental Studies and English language teaching.

### Data collection

The study collected data through two phases. The first phase involved administering a questionnaire and the second phase involved conducting a semi-structured face-to-face interview.

#### Phase 1: A questionnaire

A structured questionnaire developed from literature with a three-point Likert scale was used to solicit relevant data. The questionnaire collected information covering: *demographic variables; components or quality indicators* of teachers’ preparedness for inclusion and *competencies* (knowledge, attitude and practical skills) to effectively implement inclusive education. A total of 173 questionnaires were administered to 152 (109 males and 43 females) respondents. The questionnaire facilitated the collection of large quantities of data over a relatively short period of time (Tshuma & Mafa 2013) and enabled the presentation of the data in numerical form that is comparatively straightforward to analyse (Creswell 2012).

#### Phase 2: Semi-structured interviews

A semi-structured interview enabled the collection of detailed information in a conversational style (Harrell & Bradley 2009). Furthermore, they afforded the flexibility to explore trends, unexpected results and significant findings that were raised during the first phase of data analysis (Creswell & Plano Clark 2011). Semi-structured interviews were conducted with seven teachers in a face-to-face manner at the place suggested by participants. To avoid disruptions to academic activities, the interviews were conducted in the afternoons. Each interview was a one-time session and lasted about 50 min – 60 min. With the participants’ permission, the interviews were audio recorded. The following two questions were explored, followed by probing where appropriate:

1. How has your university facilitated your preparation for inclusion?
2. In what ways does the preparation contribute to the development of core competencies essential for inclusion?

### Data analysis

Qualitative and quantitative data were analysed separately using two different methods. Quantitative data were analysed statistically using SPSS version 6. Subsequently, descriptive (frequency, percentage, mean and standard deviation) and inferential statistics (specifically multiple regression) were applied to derive meaningful insights. The analysis facilitated a nuanced exploration of the intricate interplay between various factors, contributing to a more comprehensive understanding of the phenomenon studied.

The analysis of qualitative data was thematic, following Braun and Clarke’s (2006) framework. The process involved reading and reading transcripts to obtain familiarity with the data and jotting notes. We developed initial codes independently guided by the research questions, and we then came together to compare our ideas, discussed our codes and refined them. We then organised the codes into broader themes, to which we assigned a tentative name. The themes and names were refined following feedback from a critical reviewer. This method facilitated the extraction of meaning from a substantial volume of data, ensuring that the analysis remained focussed (Creswell & Plano Clark 2011).

The analysis of both quantitative and qualitative data was then synthesised to present a concise and accessible summary of major research findings presented in the ‘Results’ section.

### Ethical considerations

The study was granted ethical clearance by the Research Ethics Committee (REC) at the authors’ institution, the University of South Africa (reference no.: 2022/06/08/55765998/06/AM). Permission to conduct the study was approved by the College of Education at the University of South Africa. This was followed by a thorough explanation of the study, research methods and research ethics, which included participants’ rights to participate voluntarily and to withdraw from the study at any time without negative consequences. Anonymity and confidentiality were clarified as well as non-payment for participation. All participants gave written consent to participate in the study and to audio-record the interviews.

## Results

The extent to which teachers feel adequately prepared to develop the competencies required for inclusive teaching is presented in three headings: knowledge-related competency, attitude-related competency, and practical skills and abilities competency. The quantitative results obtained through the questionnaire are organised into tables; frequency and percentage are also computed. Qualitative data are used to complement the quantitative findings of the study. The seven participants are coded using pseudonyms (TA, TB, TC, TD, TE, TF and TG) to guarantee their anonymity and confidentiality for the qualitative part.

### Knowledge-related competency

Table 1 illustrates the positive outcomes from the training. Specifically, 67.2% and 71.1% of the respondents reported that the training equipped them with knowledge of national and international policies, legal frameworks, as well as local institutional regulations that effectively promote and implement inclusion. Additionally, 67.8% and 75.7% of the respondents agreed that the training helped them understand the principles and methods of teaching, along with the psychological and behavioural characteristics of diverse students. It also helped them in understanding how to assess learners with diverse needs, the practices related to inclusion and the associated issues, along with the theories that guide the implementation of inclusion (72.3%, 73% and 74.4%). Furthermore, 68.4%, 71% and 73% of the respondents believed that the training equipped them with knowledge related to addressing diversity, fostering the belief that all children can learn with appropriate support and understanding the importance of collaboration with various stakeholders in the implementation of inclusion.

**TABLE 1:** Knowledge-related competency developed as a result of the Postgraduate Diploma in Teaching programme.

No.	The PGDT programme supported me to …	Disagree		Undecided		Agree	
		*f*	%	*f*	%	*f*	%
1	… know international policies and legal frameworks	36	23.6	14	9.2	102	67.2
2	… know the principles and methods of teaching	32	20.0	17	11.2	103	67.8
3	… understand the psychological characteristics	20	13.1	17	11.2	115	75.7
4	… know how to assess students with disabilities	26	17.1	16	10.5	110	72.3
5	… know the practices associated with inclusion	27	17.7	14	9.2	111	73.0
6	… understand the theories and principles guiding inclusion	24	15.8	15	9.9	113	74.4
7	… know national policies, legal frameworks and institutional regulations	26	17.1	18	11.8	108	71.1
8	… understand how to address diversity in schools	29	19.1	19	12.5	104	68.4
9	… understand that all students have the capacity to learn and develop if properly supported	26	17.1	18	11.8	108	71.0
10	… know how to actively negotiate with educational leaders at various levels	28	18.5	13	8.6	11	73.0

PGDT, Postgraduate Diploma in Teaching.

Notably, all the percentage scores in the study fell within the 60% to 79% range, which suggests that the PGDT programme has a moderately positive impact on enhancing teachers’ knowledge in accordance with Bowen and Power’s (2005) criteria. However, the responses of interviewees varied. For example:

‘I faced challenges to understanding and support a student with learning problem in my class, because I do not know the details of what learning disability is, and how such kind of students can be supported. Even if “Peda” training is good … there was not enough time to practice what we have learned.’ (Participant 3, TC: 26 years, Male and holder of BSc [Mathematics] degree)

‘I feel that “Peda” did not give me ample opportunity to understand the expectations and culture of the surrounding community; school settings and guidelines, and factors that may affect classroom instruction in this region.’ (Participant 1, TA: 24 years, Male and holder of BSc [Sport Science] degree)

‘The “Peda” training I have taken didn’t helped me in developing my confidence and making me realise the wish to be involved in teaching of disabled students because of the very little knowledge I got from Peda.’ (Participant 5, TE: 27 years old, male and holder of BSc [Physics] degree)

‘I believe that every one of us has different capacity to learn; the problem is that I am not capable to properly support and cultivate the diverse ability and potential of my students. I feel that the “Peda” training did not give me enough knowledge with different approaches to support my students.’ (Participant 7, TG: 24 years, Male and holder of BSc [Chemistry] degree)

‘From “Peda” training, I observed that even lecturers have knowledge gap on how to handle students with disability; they can’t be role models for us. I feel that more knowledge on handling students with disability would have been passed to us. (Participant 2, TB: 23 years old, female and holder of BSc [Biology] degree)’

In contrast, two of seven interviewees stated that they have gained sufficient knowledge about inclusive teaching and students with disabilities:

‘I have got adequate awareness and understanding on the educational and social problems and issues that can affect students’ learning in secondary schools; and I have developed teaching strategies that can I use to support and deal with such difficulties in an inclusive classroom from the “Peda” training.’ (Participant 4, TD: 22 years, Female and a holder of BA [Geography and Environmental Studies] degree)

‘I have got a good grasp of most-up-to-date national policies and local regulations of inclusive education in Ethiopia as well as I have adequate understanding about the psychological, physical, and educational needs of secondary school level students from the inclusive education course I have taken during the training.’ (Participant 6, TF: 25 years old, female and holder of BA [English Language] degree)

Qualitative data confirmed that the programme had a moderate level of effectiveness in providing teachers with knowledge on inclusive education, learners with special needs and disabilities, and relevant legal and policy frameworks, and strategies from national and international perspectives.

### Attitude related competency

Table 2 illustrates that a significant proportion of respondents (65.8% and 67.1%) believe that learners with disabilities are entitled to receive quality education within inclusive settings. Additionally, 67.8%, 76.3% and 76.3% of respondents expressed that inclusion provides social benefits for learners with disabilities. These findings underscore the importance of fostering positive attitudes towards learning environments that promote inclusivity and a sense of belonging for learners with disabilities.

**TABLE 2:** Attitude-related competency developed as a result of the Postgraduate Diploma in Teaching programme.

No.	After PGDT training, I believe that …	Disagree		Undecided		Agree	
		*f*	%	*f*	%	*f*	%
1	… all students are entitled to receive quality education	41	27.0	11	7.2	100	65.8
2	… inclusion makes all students more confident	24	15.8	25	16.4	103	67.8
3	… inclusive teaching provides good opportunity for all students	32	21.1	18	11.8	102	67.1
4	… inclusion helps all students to socialise	22	14.5	14	9.2	116	76.3
5	… inclusive settings help to reduce social discrimination	27	17.7	9	5.9	116	76.3
6	… through inclusion, all students have the opportunity to improve their learning	27	17.8	16	10.5	109	71.8
7	… inclusion helps teachers to give attention for individual differences	34	22.4	13	8.6	105	69.0
8	… inclusion urges schools to improve education quality	26	17.1	15	9.9	111	73.0
9	… inclusion requires all teachers to work in teams	29	19.1	9	5.9	114	74.0
10	… inclusion requires parental and community support	29	19.1	12	7.9	111	73.0

PGDT, Postgraduate Diploma in Teaching.

Teachers believe that inclusion enhances the learning of students with disabilities, promotes a heightened awareness of individual differences, encourages school reform and improves overall educational quality. It also necessitates teacher-parent collaboration, as well as community support (71.8%, 69%, 73%, 74% and 73%). The data also suggest that the PGDT programme is likely to have a significant influence on the cognitive and affective aspects of teachers’ attitudes. However, the predisposition towards practical action may have a lower probability of being influenced by the PGDT programme because of the existing gap between theory and practice.

Therefore, the PGDT programme demonstrates a moderate level of effectiveness in positively shaping teachers’ attitudes towards inclusion. This conclusion is drawn from the percentage scores for the programme’s impact, falling within the range of 60% and 79% for all items as outlined by Bowen and Power’s (2005) criteria for assessing attitude development using a Likert scale. Despite the programme’s positive influence on the cognitive and affective aspects of attitudes, there appears to be a gap in translating these attitudes into practical behavioural actions. The interview responses vary. For instance:

‘To be honest, I don’t believe that I have the capacity to make a difference to the learning of students with disability; it is a responsibility of special educator to teaching students with disability.’ (Participant 5, TE: 27 years old, Male and BSc [Physics] degree)

‘As far as I am concerned, I believe that it is impossible and very difficult to include students with vision problems to mathematics class; rather they are excluded and stay outside.’ (Participant 3, TC: 26 years, Male and holder of BSc [Mathematics] degree)

‘I feel that the inclusion of students with disability cannot be beneficial for others normal students. This is because, students with disability will monopolise the teachers’ time and difficult to give appropriate attention to all students.’ (Participant 1, TA: 25 years, Male and holder of BSc [Sport Science] degree)

‘I feel uncomfortable and more stressed around students with disability in my school. Including students with disability into regular class create extra work for teachers like me who don’t have experience of handling such kind of cases.’ (Participant 6, TF: 25 years old, female and holder of BA [English Language] degree)

‘In our culture, the society does not support students with disability believing that their disability comes from God and if we support them, we fear that the impairment may go with us to our homes. Such kind of thinking and believe also has an influence on students with disabilities.’ (Participant 4, TD: 22 year old, Female and holder of BA [Geography and Environmental Studies] degree)

On the contrary, two out of seven participants reacted positively towards the role inclusive education plays in helping students with disabilities. For example:

‘I believe that students with disability worth educating and they have capacity to learn if they are properly supported; doing this is not only the task of special educators, but also regular teachers are responsible to teach, socialise and support students with disability.’ (Participant 2, TB: 23 years old, Female and holder of BSc [Biology] degree)

‘As a teacher, the most important thing is to be sympathetic to students with disability in and outside the classroom. I am very positive and trying my best to include such kinds of students into class activities, rather than exclude them or ignore them.’ (Participant 7, TG: 24 years, Male and holder of BSc [Chemistry] degree)

Both quantitative and qualitative results highlight the importance of influencing teacher’s attitudes, especially during teacher education training.

### Practical skills and abilities related competency

Table 3 suggests that graduates have developed the necessary skills to facilitate collaborative learning among learners with disabilities, as well as the capacity to provide differentiated instruction to students with disabilities (58.6% and 51.3%). However, while some teachers felt that the training programme supported the development of practical skills for collaborative work with other professionals teaching students with disabilities, about 50% believed that their programmes adequately prepared them for designing adaptable coursework and creating individualised assessments. Moreover, 51% to 65.2% felt that their training only moderately or minimally supported them in acquiring the skills related to adjusting teaching objectives, organising group discussions and collaborating to address the specific needs of students with disabilities.

**TABLE 3:** Practical skills-related competency developed as a result of the Postgraduate Diploma in Teaching programme.

No.	The PGDT programme enabled me to …	Disagree		Undecided		Agree	
		*f*	%	*f*	%	*f*	%
1	… make students all help and learn from each other	41	27.00	22	14.4	89	58.6
2	… conduct differentiated teaching	33	21.78	41	27.0	78	51.3
3	… work collaboratively with teachers and professionals	44	28.90	24	15.8	84	55.3
4	… design flexible coursework and individual assessment	47	30.90	29	19.1	76	50.0
5	… adjust teaching objectives accordingly	33	21.70	40	26.3	79	51.9
6	… arrange group work and collaborative learning	37	24.30	16	10.5	99	65.2
7	… conduct effective behavioural management	35	23.10	39	25.6	78	51.3
8	… work effectively with parents	46	30.30	25	16.4	81	53.3
9	… work effectively with the communities	39	25.70	30	19.7	83	54.6
10	… use various teaching and learning materials	35	23.10	40	26.3	77	50.6

PGDT, Postgraduate Diploma in Teaching.

Table 3 also shows that teachers have minimally developed practical skills and abilities required for inclusive education (60%). These skills include conducting effective behavioural management, working with parents, engaging with communities and using various teaching materials to support students with disabilities. This indicates a need for improvement in the programme’s preparation of teachers for inclusive teaching.

In this regard, six out of the seven interviewees responded that they did not have the skills and abilities necessary to make a difference in the lives of students with disabilities. It seems that the PGDT programme did not fully support the teachers in gaining the necessary skills to implement inclusive education successfully in Ethiopia. For instance:

‘During the teaching practices, I have not done much to implement inclusive teaching because I did not cope with the diversity within the classroom, and time allocated for a single lesson was also 50 minutes, which is difficult to manage as a fresh teacher; I feel that I need further skills that can help me to adapt a lesson and address students with disabilities in the classroom and also outside of the classroom as I am sport science teacher.’ (Participant 1, TA: 25 years, Male and holder of BSc [Sport Science] degree)

‘Our lecturers agreed with the teacher education college timetable, which was too loaded, as a result, they face difficult to effectively equip us with the necessary inclusive skills and abilities; I don’t feel confident …, uhu … I found it very challenging to have a student with disability in my class.’ (Participant 2, TB: 23 years old, Female and a holder of BSc [Biology] degree)

‘We are required to teach students with and without disabilities at the same time in the same classroom. We need different strategies to meet the needs of different students; we need to organise group activities in classroom that include all students, which requires different skills again. We need to think how to get students with disabilities engage in learning. Do you think that I have gained all those skills from “peda” training? No, to be honest, it is very difficult to manage students with disabilities.’ (Participant 3, TC: 26 years old, Male and a holder of BSc [Mathematics] degree)

‘Behaviour problem is very common in school I assigned, it overwhelmed me at first and I didn’t know what to do. I think I missed important skill that helps me to manage students’ behaviour in classroom. I feel that “Peda” training is full of theory, I suggest being more practical during the training, because you can read and understand the theory part. The practical part of teaching needs more engagement.’ (Participant 4, TD: 22 years, Female and a holder of BA [Geography and Environmental Studies] degree)

The findings suggest that the PGDT programme has not adequately equipped teachers with the necessary practical skills and competencies for inclusive teaching. This gap points to the need for teacher education to provide teachers with practical skills and hands-on experiences that can address the learning needs of students. The analysis of these findings is presented in the ‘Discussion’ section.

## Discussion

Based on the findings of the present study, one can safely argue that the teacher training programme in Ethiopia does not adequately equip teachers with sufficient knowledge about inclusion and students with disabilities. This finding finds resonance in the EMoE’s (2018) report, which indicated that Ethiopian teachers generally have limited to no understanding of inclusion and specific needs of students with disabilities. This challenge is not unique to Ethiopia, as studies in Malaysia, Greece and other parts of Africa have also highlighted similar shortcomings in teacher training. For example, Alias and Salleh’s (2017) study conducted in Malaysia revealed that teachers often lacked the requisite training with respect to understanding learners’ characteristics and needs, appropriate teaching aids and effective strategies to be employed. Similarly, Akalin et al.’s (2014) study found that teachers in Greece faced challenges related to limited knowledge about inclusive practices and a lack of clarity regarding their roles in inclusive settings. Recently, Naami and Saa-Touh Mort (2023) found that disability was notably absent from the curriculum, learning outcomes of the equity and inclusivity cross-cutting pillar, their targets and supplementary sources used in the colleges of education in Ghana. In South Africa, Nseibo et al. (2022) found that initial teacher education had little focus on teaching and supporting learners with disabilities, especially those with severe and profound disabilities despite the country’s ratification of numerous human rights agreements that support the provision of education for all learners and the educational policies that require that teachers boost competency in these areas. Participants in Majoko and Phasha’s (2018) study complained about a lack of exposure to relevant theories of inclusive education, superficial exposure to policy, and a lack of opportunities to teach learners with disabilities. These findings collectively highlight the global importance of enhancing teacher preparation programmes to better support the principles of inclusive education.

The 2009 curriculum framework in Ethiopia stipulates that teachers should have adequate knowledge about inclusion to enable them to identify and assess the diverse needs, challenges and potentials of students. This is in alignment with inclusive pedagogy, which requires teachers to be professionally knowledgeable to effectively engage with diverse students, especially in this era of inclusion (Florian 2016). They are also required to have an awareness of the learners’ socio-cultural and socio-political contexts (UNESCO 2009). Therefore, the lack of adequate preparation in terms of knowledge necessary for implementing inclusion suggests that many teachers in Ethiopia may face challenges in accommodating and addressing diversity. As noted by Kortjass (2012) and West and Hudson (2010), teachers with limited understanding of inclusion, may resist the invitation to develop a more inclusive school.

Inadequate teacher training demonstrates Mu et al.’s (2015) and Johnstone and Chapman’s (2009) point about the critical role teacher education programmes play in the successful implementation of inclusive education. These include raising awareness about inclusion among teachers so that, after training, they are equipped to support all learners in order for them to reach their full potential (Hodgson & Wilkerson 2013). Also, it should provide teachers with essential knowledge organised in a way that would enable them to develop a deep understanding of inclusive pedagogy (LePage et al. 2010).

The finding that the PGDT demonstrated moderate effectiveness in developing teachers’ attitudes towards inclusion and students with disabilities gives rise to doubts about its overall impact on shaping these attitudes. It also raises questions about the teachers’ ability to create inclusive schools and society. In light of Loreman et al.’s (2013) point that teacher education can influence attitudes, the programmes must strive to close the gaps between theory and practice as suggested by Munyungu (2015). In addition, it should provide opportunities for teachers to engage with diverse learners and become familiar with policies and legislation related to inclusive education. These components are equally important in the process of developing positive attitudes towards inclusion.

The current PGDT in Ethiopia faces a challenge, as its modules and courses are overly theoretical and too reliant on the medical model of disability, which promotes negative perceptions of disability (Zelalem 2021). As highlighted by Galović, Brojčin and Glumbić (2014), the adherence to the medical model contributed to teachers’ development of negative attitudes towards inclusion and students with disabilities in Ethiopia, leading to determinist beliefs about children’s worth and abilities. Notably, this approach has hindered the efforts of Ethiopian teacher education institutions in creating supportive and inclusive learning environments for students with disabilities (Tefera, Adams & Mulatie 2015).

Hart et al. (2004) argued that inclusive pedagogy begins with the notion that all learners are different and that human diversity should not be neglected or disregarded. Therefore, learning should be based on positive differentiation, which contrasts segregation and mindless inclusion. In essence, teachers need to accept and recognise individual differences as a natural aspect of the human condition in the classroom (Rouse 2010) and overcome any constraints imposed by deterministic beliefs (Hart et al. 2004). Therefore, it is important to address the complexity of teachers’ attitudes towards inclusion and students with disabilities during teacher training (Florian & Spratt 2013). The training should be well-structured and include hands-on courses and assignments (Berisha & Vula 2021). Modules and courses should help teachers understand the environmental, social and cultural contexts of learning and teaching (eds. Alur & Timmons 2009). Additionally, it should expose teachers to various pedagogical approaches (Pather 2019), and equip them with the competencies to navigate the complex and multifaceted environment of an inclusive classroom (EMoE 2009). This will ensure that effective teaching and learning, along with appropriate support strategies, are accessible in inclusive educational settings (Florian & Black-Hawkins 2011).

Such training will not only benefit learners with diverse educational needs but also contribute to the broader goal of establishing an inclusive society. However, ill-prepared teachers who lack the fundamental skills and abilities necessary for inclusion, often experience dissatisfaction, which may inadvertently affect students, potentially undermining the confidence and overall success of learners with disabilities (Jordan, Schwartz E. & McGhie-Richmond 2009). The ‘Conclusion’ section presents the final findings and recommendations.

## Conclusion

In this article, the analysis of the effectiveness of a teacher education programme in Ethiopia in developing teachers’ competencies for inclusion is presented. As the findings of the present study have shown, teacher training in Ethiopia is still lagging behind in terms of keeping pace with global changes that support teachers in developing essential competencies for the successful implementation of inclusive education. The current system falls short in providing teachers with a sufficient understanding of inclusion, developing positive attitudes and adequately exposing them to practical, hands-on experience. This shortcoming impedes the equitable delivery of quality education. To address this, substantial changes in policies and practices are necessary, including an overhaul of the teacher education curricula, fostering positive attitudes and improving practical learning experiences for teachers. These improvements will ensure that teachers are well-prepared to cater to the diverse needs of learners and establish an inclusive learning environment.

This study has limitations, one of which is its focus on a single university in Ethiopia, excluding other important stakeholders who could offer perspectives different from those of teachers. To gain a more comprehensive understanding, future research should encompass all 10 universities in Ethiopia and involve a broad range of participants. Such an approach will provide diverse perspectives and insights to better advance teacher preparation for inclusive education in Ethiopia.
